# Robot-Assisted Versus Open and Laparoscopic Radical Nephrectomy with Inferior Vena Cava Thrombectomy for Renal Cell Carcinoma: A Systematic Review and Meta-Analysis

**DOI:** 10.3390/cancers18162613

**Published:** 2026-08-13

**Authors:** Filippo Caudana, Mattia Ronca, Francesco Ditonno, Greta Pettenuzzo, Celeste Manfredi, Alessandro Veccia, Riccardo Giuseppe Bertolo, Gaëlle Margue, Riccardo Autorino, Jean-Christophe Bernhard, Alessandro Antonelli

**Affiliations:** 1 Department of Urology, Azienda Ospedaliera Universitaria Integrata of Verona, University of Verona, 37126 Verona, Italy; filippo.caudana@aovr.veneto.it (F.C.); mattia.ronca@aovr.veneto.it (M.R.); greta.pettenuzzo@aovr.veneto.it (G.P.); alessandro.veccia@aovr.veneto.it (A.V.); riccardogiuseppe.bertolo@univr.it (R.G.B.); alessandro.antonelli@aovr.veneto.it (A.A.); 2 Urology Department, Bordeaux University Hospital, 33076 Bordeaux, France; gaelle.margue@chu-bordeaux.fr (G.M.); jean-christophe.bernhard@chu-bordeaux.fr (J.-C.B.); 3 I.CaRe Bordeaux—BRIC Inserm, UMR1312, Bordeaux Institute of Oncology, Bordeaux University, 33076 Bordeaux, France; 4 Unit of Urology, Department of Woman, Child and General and Specialized Surgery, University of Campania “Luigi Vanvitelli”, 80138 Naples, Italy; celeste.manfredi@unicampania.it; 5 Glickman Urological Institute, Cleveland Clinic, Cleveland, OH 44195, USA; autorir@ccf.org

**Keywords:** carcinoma, renal cell, vena cava, inferior, thrombectomy, robotic surgical procedures, nephrectomy

## Abstract

Background/Objectives: To compare perioperative, pathological, functional, and oncological outcomes of robot-assisted radical nephrectomy with inferior vena cava tumor thrombectomy (RARN-TT) versus open (ORN-TT) and laparoscopic (LRN-TT) approaches. Methods: PubMed, Scopus, and Web of Science were searched for studies of adults with renal cell carcinoma and Mayo/Neves level I–IV inferior vena cava tumor thrombus undergoing RARN-TT versus ORN-TT and/or LRN-TT. Risk ratios and mean differences with 95% confidence intervals were calculated using random effects models with restricted maximum-likelihood estimation. Results: Eight retrospective studies including 1781 patients were included: 221 underwent robotic surgery, 1411 open surgery, and 149 laparoscopic surgery. Compared with ORN-TT, RARN-TT was associated with lower estimated blood loss (mean difference −900.5 mL, 95% confidence interval −1234.0 to −566.9; *p* = 0.001), lower transfusion probability (risk ratio 0.395, 95% confidence interval 0.159–0.979; *p* = 0.046), and shorter hospital stay (mean difference −3.79 days, 95% confidence interval −4.83 to −2.76; *p* < 0.001). No significant differences were observed in operative time, intensive care unit stay, postoperative complications, perioperative mortality, pathological outcomes, or overall survival. Evidence for cancer-specific and progression-free survival was limited. Comparisons with LRN-TT were exploratory. Conclusions: RARN-TT may reduce blood loss, transfusion requirements, and hospital stay compared with ORN-TT, without evidence of worse perioperative, pathological, or survival outcomes. However, all studies were retrospective and affected by selection bias and heterogeneity. RARN-TT may be considered for selected patients at experienced centers, particularly for lower-level thrombi. Prospective multicenter studies stratified by thrombus level are needed.

## Simple Summary

Kidney cancer can sometimes grow into the inferior vena cava, making surgery complex and risky. Open surgery has traditionally been the standard treatment, but robot-assisted and laparoscopic techniques are increasingly used in specialized centers. This systematic review and meta-analysis compared these approaches to determine whether minimally invasive surgery offers benefits without compromising cancer control. Eight retrospective studies including 1781 patients were analyzed. Robotic surgery was associated with less blood loss, fewer blood transfusions, and a shorter hospital stay than open surgery. No clear differences were found in operative time, complication rates, pathological findings, or survival outcomes. However, the evidence was limited by patient selection, differences between centers, and the lack of randomized studies. These findings support robotic surgery as a possible option for carefully selected patients in experienced centers, while further high-quality studies are needed, especially for more advanced tumor thrombi.

## 1. Introduction

Radical nephrectomy with inferior vena cava (IVC) tumor thrombectomy represents the standard surgical treatment for renal cell carcinoma (RCC) with venous tumor thrombus. This condition occurs in approximately 4–10% of patients with RCC, creating a technically demanding scenario associated with substantial perioperative risk [1,2]. Open surgery has traditionally been the reference approach, especially for complex or high-level thrombi, because it provides direct exposure and better vascular control [2,3]. However, it is associated with relevant perioperative morbidity and postoperative complications [2,3,4]. Minimally invasive approaches have achieved widespread adoption across major urological procedures and are progressively gaining momentum even in this highly complex scenario [1,5]. In this context, while laparoscopic thrombectomy remains technically challenging, the robotic platform overcomes these limitations by facilitating dissection, vascular control, and reconstruction through enhanced visualization, dexterity, and intracorporeal suturing [1,6,7]. However, the robotic systems evaluated in the available studies lacked native force feedback, requiring surgeons to rely predominantly on visual cues; within the da Vinci platform, integrated force feedback became available with the da Vinci 5, introduced in 2024 [8,9]. Available comparative evidence suggests potential perioperative advantages for robot-assisted surgery in selected patients, particularly for Mayo level I–II thrombi, while data for level III–IV thrombi remain limited and mostly derived from expert centers [4,6,7,10]. However, the evidence is limited by retrospective designs, selection bias, heterogeneous reporting, and incomplete oncological follow-up [1,2].

Building on these premises, the present systematic review and meta-analysis aim to compare robot-assisted radical nephrectomy with IVC thrombectomy (RARN-TT) with open (ORN-TT) and laparoscopic (LRN-TT) approaches in adult patients with RCC and venous tumor thrombus classified according to either the Mayo or the Neves–Zincke system [11,12,13].

## 2. Materials and Methods

The study protocol was registered in the International Prospective Register of Systematic Reviews database (PROSPERO: CRD420251153731); no amendments were made to the registered protocol.

### 2.1. Protocol and Registration

The present systematic review and meta-analysis was performed according to the Preferred Reporting Items for Systematic Reviews and Meta-Analyses 2020 statement [14] and the Meta-Analysis Of Observational Studies in Epidemiology (MOOSE) recommendations for observational evidence [15], in line with contemporary guidelines for urology meta-analysis [16]. A systematic literature search was conducted through MEDLINE^®^, Scopus^®^, and Web of Science™ in June 2026.

The research question was structured according to the PICO framework: adult patients with renal cell carcinoma (RCC) and inferior vena cava (IVC) tumor thrombus (P), undergoing robot-assisted radical nephrectomy with IVC thrombectomy (RARN-TT) (I), versus open radical nephrectomy with IVC thrombectomy (ORN-TT) and/or laparoscopic radical nephrectomy with IVC thrombectomy (LRN-TT) (C), to assess perioperative, pathological, renal functional, and oncological outcomes (O), across comparative studies (Studies).

The full search strings for each database are provided in the Supplementary Material.

Reference lists of included studies and relevant reviews were manually screened to identify additional eligible articles [17,18].

### 2.2. Eligibility Criteria

**Figure 1:**
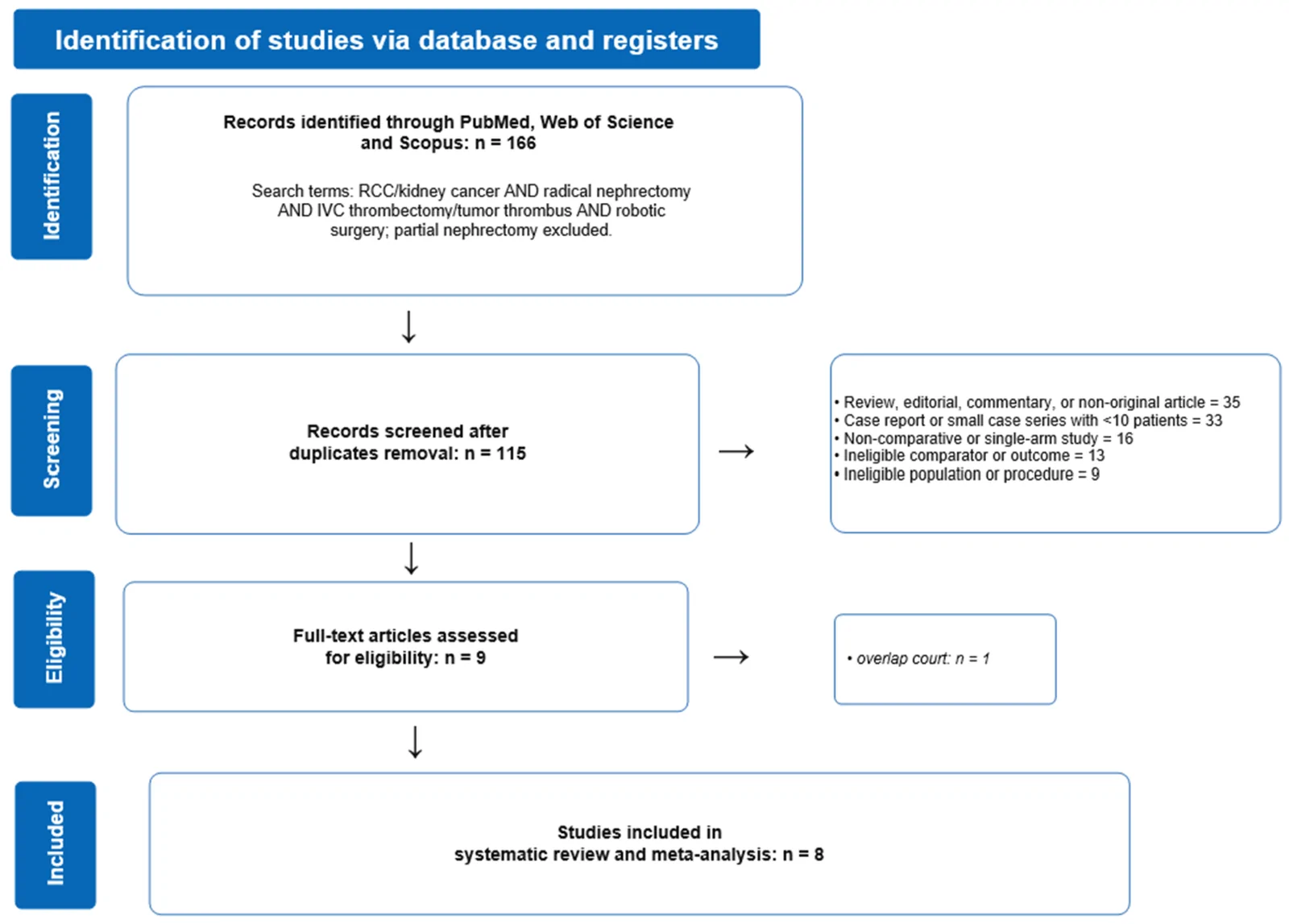
Literature search process. PRISMA flow-chart for study selection.

After duplicate removal, titles and abstracts were independently screened by two reviewers (G.P., M.R.). Full-text articles were assessed when eligibility could not be determined from title and abstract alone. Disagreements were resolved by consensus or by consultation with a third reviewer (F.D.). The selection process was summarized using a PRISMA flow diagram (Figure 1).

Eligible studies included prospective or retrospective cohort studies, case–control studies, and randomized controlled trials enrolling adult patients aged ≥18 years with histologically confirmed RCC and Neves/Mayo level I–IV IVC tumor thrombus treated with radical nephrectomy and IVC thrombectomy. Studies were required to compare RARN-TT with ORN-TT and/or LRN-TT and to report at least one predefined perioperative, renal functional, pathological, or oncological outcome.

Clinical trial protocols, reviews, editorials, conference abstracts, non-original articles, non-comparative studies, single-arm case series, case reports, studies including fewer than 10 patients, studies evaluating partial nephrectomy, studies with ineligible comparators, and studies without predefined outcomes were excluded. In case of duplicate or overlapping cohorts, the most complete and methodologically appropriate report was selected, prioritizing the largest cohort, the most recent dataset, and the most comprehensive outcome reporting.

### 2.3. Information Sources and Search Strategy

Data were independently extracted by two reviewers (F.C., C.M.) using a standardized extraction form. Discrepancies were resolved by consensus or by consultation with a third reviewer (A.A.). Extracted study-level variables included first author, year of publication, country, study design, study period, surgical approach, sample size, eligibility criteria, and follow-up duration. Patient- and tumor-level variables included age, sex, body mass index (BMI), comorbidity status, performance status when available, tumor laterality, tumor size, histological subtype, tumor grade, pathological stage, nodal status, metastatic status, thrombus level, thrombus length, IVC wall invasion, neoadjuvant systemic therapy, and cardiopulmonary bypass use when reported. Thrombus classification was recorded as originally reported by each study, and no conversion between the Mayo and Neves–Zincke systems was performed. Perioperative outcomes included operative time (OT), estimated blood loss (EBL), conversion to open surgery, transfusion rate, postoperative complications, major postoperative complications, length of hospital stay (LOS), intensive care unit stay and perioperative mortality.

Major postoperative complications were defined as Clavien–Dindo grade ≥III when compatible grading was available. Oncological outcomes included OS, CSS, RFS, PFS, MFS, LRFS, disease progression, recurrence, and death, as defined and reported by each included study. For time-to-event outcomes, HRs with 95% CIs were preferentially extracted from multivariable models, followed by univariable estimates or Kaplan–Meier-derived data when available. When survival estimates were not directly reported, approximate individual patient data were reconstructed from published Kaplan–Meier curves and numbers at risk using the Guyot algorithm, and the corresponding time-to-event outcomes were derived from the reconstructed data. When HRs could not be directly extracted or reliably derived, survival outcomes were not included in quantitative synthesis and were summarized narratively.

### 2.4. Statistical Analysis

A narrative synthesis was provided for all included studies. A meta-analysis was performed when at least two clinically and methodologically comparable studies reported the same outcome. Analyses were conducted using Stata 19.0 (StataCorp LLC, College Station, TX, USA). Given the anticipated between-study variability, we used random-effects models with restricted maximum likelihood (REML) estimation of between-study variance and applied Hartung–Knapp adjustments for uncertainty in the pooled effect where appropriate (particularly with small numbers of studies).

Risk ratios with 95% confidence intervals were calculated for dichotomous outcomes. Mean differences with 95% confidence intervals were calculated for continuous outcomes; standardized mean differences were planned when direct pooling was not appropriate. Hazard ratios with 95% confidence intervals were planned for time-to-event outcomes when sufficiently consistent and extractable data were available.

Heterogeneity was evaluated primarily through inspection of forest plots and reporting of τ^2^ alongside prediction intervals to communicate the plausible range of effects in future settings. Cochran’s Q test was reported as a descriptive measure of statistical heterogeneity. Outcomes reported by only one study were summarized descriptively and were not pooled. Because fewer than 10 studies contributed to each outcome, subgroup analysis and meta-regression were not performed.

### 2.5. Risk of Bias and Publication Assessment

Risk of bias was assessed independently by two reviewers (A.V., R.B.) using ROBINS-I for non-randomized studies [19]. If randomized controlled trials were identified, the Cochrane RoB 2 tool was planned [20]. Disagreements were resolved by consensus or by consultation with a third reviewer (J.C.B.). Domain-level and overall judgments were summarized for each included study. Small-study effects were explored using Egger’s regression test and Begg’s rank-correlation test. For dichotomous outcomes, the Harbord test was additionally performed. Tests were conducted only when at least three studies contributed to the analysis. Because fewer than 10 studies were available for every outcome, all analyses of small-study effects were considered exploratory and were interpreted cautiously. Given the observational design of all included studies, the small number of studies contributing to each outcome, and the heterogeneity observed for several comparisons, a formal assessment of the certainty of evidence (GRADE) was not undertaken.

## 3. Results

### 3.1. Study Selection

The database search identified 166 records through PubMed/MEDLINE, Scopus, and Web of Science. After the removal of 51 duplicates, 115 records underwent title and abstract screening. Of these, 106 were excluded because they were reviews, editorials, commentaries, or other nonoriginal articles (*n* = 35); case reports or case series including fewer than 10 patients (*n* = 33); noncomparative or single-arm studies (*n* = 16); studies with an ineligible comparator or outcome (*n* = 13); or studies evaluating an ineligible population or procedure (*n* = 9).

Nine full-text articles were assessed for eligibility. One article, reporting a monocentric cohort subsequently superseded by a larger multicentric series retained for synthesis [2], was excluded because of cohort overlap, leaving eight studies for qualitative and quantitative synthesis. The study selection process is presented in Figure 1.

A total of eight retrospective comparative studies including 1781 patients was included. Overall, 221 patients underwent robotic surgery, 1411 underwent open surgery, and 149 underwent laparoscopic surgery (Table 1).

### 3.2. Patient Characteristics

Table 2 summarizes the main baseline characteristics of patients according to surgical approach. Detailed study-level data and measures of between-study heterogeneity are reported in Table S1.

Baseline comparability was assessed according to the available data. Between RARN-TT and ORN-TT, male sex distribution was comparable (RR 0.997, 95% CI 0.884–1.125; *p* = 0.961), as were age (MD +1.19 yr, 95% CI −0.86 to +3.25; *p* = 0.212), BMI (MD +0.91 kg/m^2^, 95% CI −0.03 to +1.85; *p* = 0.056), Charlson Comorbidity Index (MD +0.60, 95% CI −8.20 to +9.40; *p* = 0.547), and ASA ≥ 3 distribution (RR 0.898, 95% CI 0.620–1.302; *p* = 0.425).

Between RARN-TT and LRN-TT, no significant differences were observed in terms of patients’ sex (RR 0.949, 95% CI 0.195–4.605; *p* = 0.744), age (MD −0.36 yr, 95% CI −22.68 to +21.97; *p* = 0.873), or BMI (MD +1.29 kg/m^2^, 95% CI −22.55 to +25.13; *p* = 0.616). High ASA class was reported for the LRN-TT group in one study only; therefore, no formal comparative analysis was possible.

### 3.3. Surgical Outcomes

Comparative surgical outcomes are summarized in Table 3. Detailed study-level results, measures of between-study heterogeneity, and prediction intervals are reported in Table S2.

**Figure 2:**
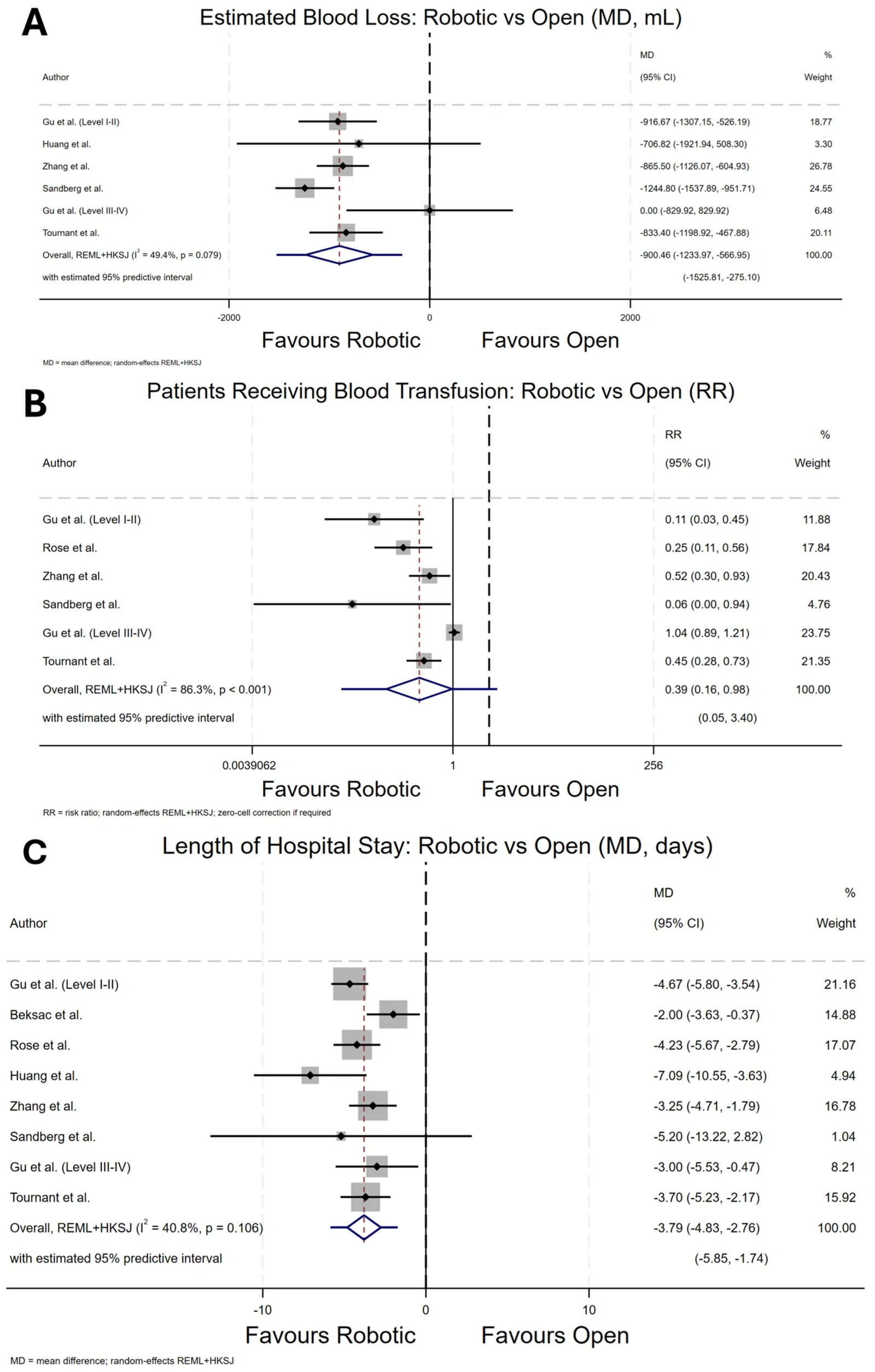
Main perioperative benefits of RARN-TT versus ORN-TT; (**A**) estimated blood loss robotic vs. open [2,3,7,10,22,23]; (**B**) patients receiving blood transfusion robotic vs. open [2,3,6,7,10,23]; (**C**) length of hospital stay [2,3,6,7,10,21,22,23].

**Figure 3:**
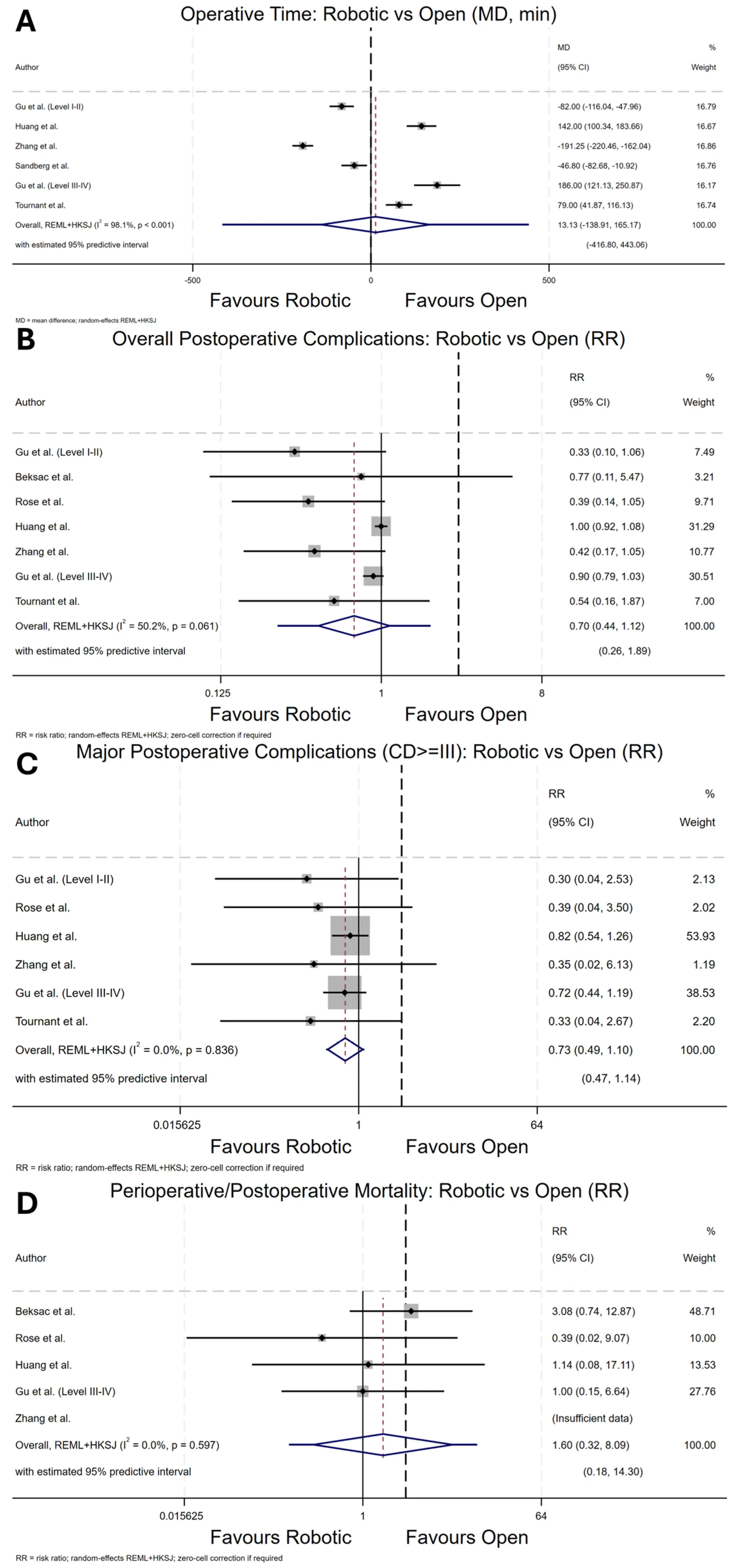
Operative time and perioperative safety; (**A**) operative time robotic vs. open [2,3,7,10,22,23]; (**B**) overall postoperative complications robotic vs. open [2,3,6,7,10,21,22]; (**C**) major postoperative complication robotic vs. open [2,3,6,7,10,22]; (**D**) perioperative/postoperative mortality robotic vs. open [6,7,10,21,22].

EBL was evaluated in the same six studies including 708 patients. RARN-TT was associated with significantly lower blood loss than ORN-TT (MD −900.5 mL, 95% CI −1234.0 to −566.9; *p* = 0.001) (Figure 2A). Although the direction of effect generally favored RARN-TT, the absolute EBL varied considerably across studies. Blood transfusion was evaluated in six studies including 686 patients. RARN-TT was associated with a lower probability of transfusion than ORN-TT (RR 0.395, 95% CI 0.159–0.979; *p* = 0.046) (Figure 2B). However, substantial between-study heterogeneity indicated that the magnitude of this association varied considerably among studies. LOS was evaluated in eight studies including 1632 patients. RARN-TT was associated with a significantly shorter hospital stay than ORN-TT (MD −3.79 d, 95% CI −4.83 to −2.76; *p* < 0.001) (Figure 2C). OT was evaluated in six studies including 708 patients. No significant difference was observed between RARN-TT and ORN-TT (MD +13.1 min, 95% CI −138.9 to +165.2; *p* = 0.833) (Figure 3A). However, the direction and magnitude of the study-specific effects varied markedly, resulting in substantial heterogeneity and limiting interpretation of the pooled estimate. Quantitative amounts of red blood cells and fresh frozen plasma were not pooled because of differences in reporting units and uncertainty regarding whether the reported averages referred to the entire cohort or only to transfused patients. Cardiopulmonary bypass use was reported in three studies, although only two provided an estimable comparative effect. No significant difference was observed between RARN-TT and ORN-TT (RR 0.605, 95% CI 0.101–3.643; *p* = 0.175). The small number of informative studies and the wide confidence interval precluded firm conclusions. IVC clamp time was reported by one study including 92 patients, with mean values of 22.2 min for RARN-TT and 13.3 min for ORN-TT. No comparative meta-analysis was therefore performed. Cardiopulmonary bypass duration could not be synthesized because no study provided complete mean, standard deviation, and sample size data for both groups. ICU stay was evaluated in three studies including 199 patients. No significant difference was observed between RARN-TT and ORN-TT (MD +0.43 d, 95% CI −5.47 to +6.32; *p* = 0.785). The study-specific effects were highly inconsistent, and the pooled estimate was therefore imprecise. Overall postoperative complications were evaluated in six studies including 429 patients. No significant difference was observed between RARN-TT and ORN-TT (RR 0.689, 95% CI 0.411–1.156; *p* = 0.124) (Figure 3B). The exclusion of Huang et al., in which all patients in both groups were classified as having a postoperative complication, did not alter the result (RR 0.570, 95% CI 0.280–1.160; *p* = 0.093). Major postoperative complications, defined as Clavien–Dindo grade ≥III, were also evaluated in six studies including 429 patients. No significant difference was observed between the approaches (RR 0.731, 95% CI 0.485–1.100; *p* = 0.106) (Figure 3C). Perioperative or postoperative mortality was reported in five studies, although one study reported no deaths in either group and did not contribute to the pooled RR. No significant difference was identified between RARN-TT and ORN-TT (RR 1.601, 95% CI 0.317–8.088; *p* = 0.424) (Figure 3D). Conversion to open surgery was reported in four RARN-TT cohorts, with a pooled conversion rate of 7% (95% CI 3–13%). Evidence comparing RARN-TT and LRN-TT was limited to two studies including 194 patients. These pooled findings should therefore be considered exploratory, while outcomes reported by one study only were considered descriptive. Operative time was reported in both studies, with no significant difference between RARN-TT and LRN-TT (MD −80.3 min, 95% CI −1147.9 to +987.3; *p* = 0.514). The two studies produced markedly discordant results, resulting in substantial heterogeneity and a highly imprecise pooled estimate. Length of hospital stay was also evaluated in both studies, with no significant difference between the approaches (MD −0.91 d, 95% CI −9.55 to +7.73; *p* = 0.408). All other surgical outcomes were reported by Zhang et al. only, including 22 patients undergoing RARN-TT and 88 undergoing LRN-TT. Mean estimated blood loss was 234.5 mL and 505.0 mL, respectively. Blood transfusion was required in 8 of 22 RARN-TT patients (36.4%) and 38 of 88 LRN-TT patients (43.2%). Overall postoperative complications occurred in 4 of 22 patients (18.2%) and 20 of 88 patients (22.7%), while major complications occurred in 0 of 22 patients and 4 of 88 patients (4.5%), respectively. No perioperative or postoperative deaths were reported in either group. Conversion to open surgery occurred in 1 of 22 RARN-TT procedures (4.5%) and 8 of 88 LRN-TT procedures (9.1%). Complete comparative data were unavailable for IVC clamp time and ICU stay.

Surgical site infection, wound dehiscence, incisional hernia, postoperative venous thromboembolism, and readmission were not reported with sufficient comparative data to permit quantitative synthesis.

### 3.4. Pathological Outcomes

Table 4 summarizes the pooled pathological characteristics according to surgical approach. Detailed study-level data, measures of between-study heterogeneity, and sensitivity analyses are reported in Table S3.

Pathological characteristics were compared according to the available data. Between RARN-TT and ORN-TT, no statistically significant differences were identified in clear-cell RCC histology across seven studies including 746 patients (OR 0.721, 95% CI 0.375–1.386; *p* = 0.267), high-grade tumors across six studies including 653 patients (OR 0.729, 95% CI 0.347–1.532; *p* = 0.323) or positive lymph nodes across three studies including 264 patients (OR 0.707, 95% CI 0.170–2.948; *p* = 0.406). IVC wall invasion and positive surgical margins were each reported by only two studies. Because the study-specific effects were discordant and the pooled comparative ORs were extremely imprecise, these estimates were considered clinically uninformative. The pooled group-specific proportions were 30% (95% CI 18–44%) versus 26% (95% CI 18–35%) for IVC wall invasion and 6% (95% CI 1–14%) versus 20% (95% CI 12–30%) for positive surgical margins in the RARN-TT and ORN-TT groups, respectively; these results are therefore presented descriptively in Table S3.

Between RARN-TT and LRN-TT, no statistically significant differences were observed in clear-cell RCC histology (OR 1.166, 95% CI 0.001–908.933; *p* = 0.818) or high-grade tumors (OR 0.795, 95% CI 0.002–261.587; *p* = 0.703). Both comparisons were based on only two studies and yielded highly imprecise estimates. LRN-TT data on IVC wall invasion and positive lymph nodes were available from one study only, while complete comparative data on positive surgical margins were unavailable; these findings were therefore considered descriptive and were not pooled.

### 3.5. Renal Functional Outcomes

Renal functional outcomes could not be analyzed because sufficiently complete and comparable data on postoperative serum creatinine, estimated glomerular filtration rate, and CKD upstaging were unavailable across the included studies.

### 3.6. Survival Outcomes

Formal meta-analysis was feasible for only three time-to-event outcomes because few studies reported extractable hazard ratios. The pooled results are summarized in Table 5, while individual study estimates for outcomes that could not be pooled are reported in Table S4.

OS was evaluated in four studies comparing RARN-TT and ORN-TT. No significant difference was observed between the approaches (HR 0.877, 95% CI 0.444–1.733; *p* = 0.583). Between-study heterogeneity was limited, although the prediction interval was wide and included potentially important effects in either direction. The result remained consistent in leave-one-out analyses and after the exclusion of Sandberg et al., for which the HR was reconstructed from Kaplan–Meier curves (HR 0.794, 95% CI 0.227–2.779). An analysis restricted to the two studies reporting direct Cox model estimates also showed no significant difference (HR 0.626, 95% CI 0.027–14.629; *p* = 0.310), although precision was very limited.

CSS and PFS were each evaluated in two studies, with no significant differences observed between RARN-TT and ORN-TT for the former (HR 1.031, 95% CI 0.009–114.401; *p* = 0.948) and the latter (HR 0.961, 95% CI 0.111–8.359; *p* = 0.855). Both estimates were highly imprecise because of the small number of contributing studies.

RFS and DFS were each reported by one study only. Neither outcome differed significantly between RARN-TT and ORN-TT, although the point estimates numerically favored the robotic approach (RFS: HR 0.440, 95% CI 0.180–1.070; DFS: HR 0.500, 95% CI 0.220–1.120).

All comparisons involving LRN-TT, as well as analyses of metastasis-free survival, were based on individual studies and showed no significant differences between surgical approaches. Several of these estimates were reconstructed from Kaplan–Meier curves and should therefore be interpreted cautiously. Detailed estimates are reported in Table S4.

Locoregional recurrence-free survival was reported by Zhang et al. only. Minimally invasive surgery was associated with improved locoregional recurrence-free survival compared with open surgery (HR 0.200, 95% CI 0.060–0.700). Because this finding originated from a single study, it was considered hypothesis-generating.

### 3.7. Risk of Bias and Publication Bias

**Figure 4:**
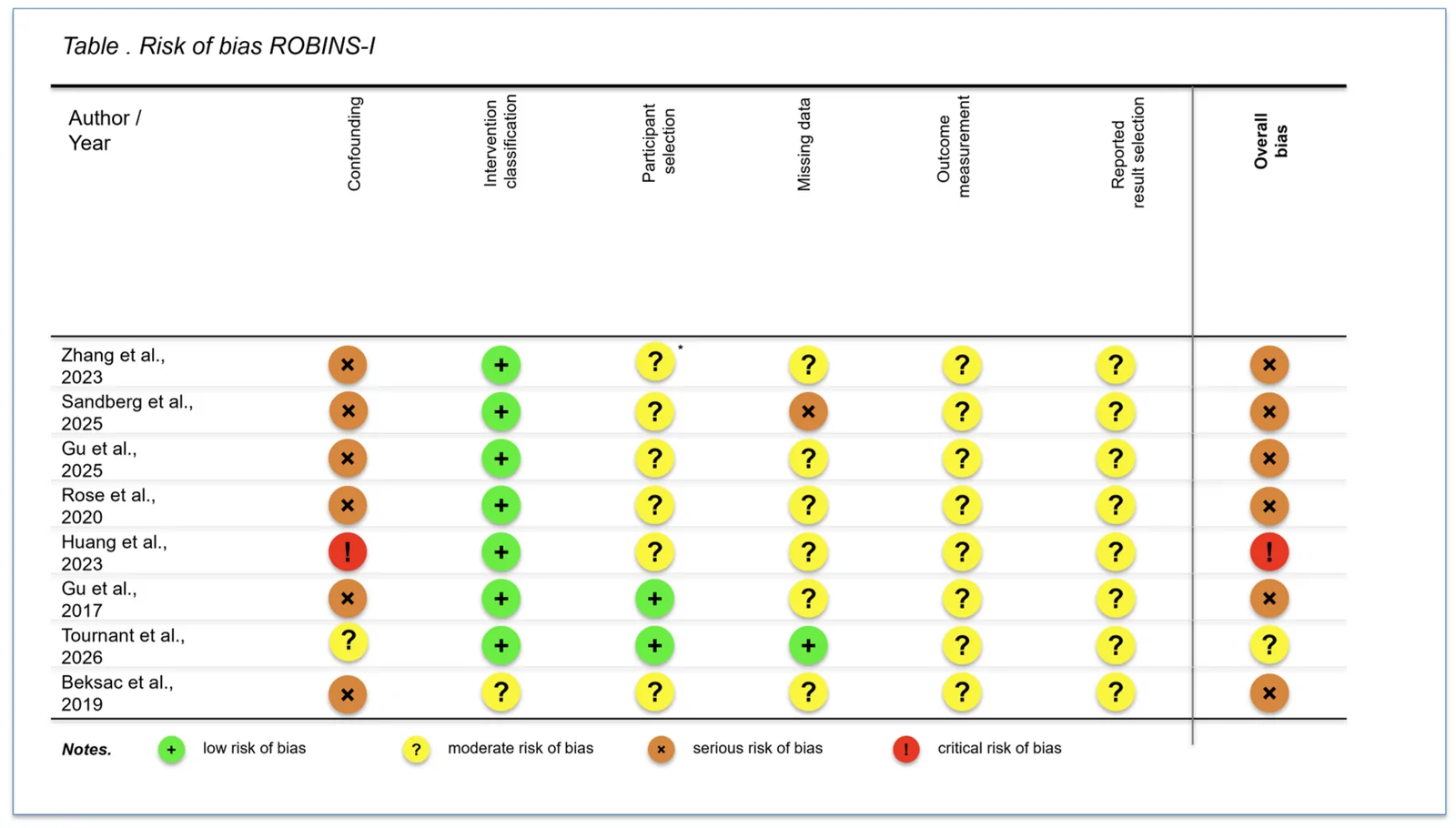
Risk of bias ROBINS-I [2,3,6,7,10,21,22,23].

Risk of bias was assessed using the ROBINS-I. Full domain-level scores per study are reported in Figure 4.

No comparison included ten or more studies. Funnel plots were therefore not interpreted, and no formal assessment of publication bias was considered reliable. Exploratory Egger and Begg tests, together with Harbord tests for binary outcomes, were nevertheless performed for analyses including at least three studies. The complete results are reported in Table S5.

## 4. Discussion

This systematic review and meta-analysis included eight retrospective comparative studies and 1781 patients to compare RARN-TT, ORN-TT and LRN-TT in terms of perioperative, pathological, and survival outcomes. Compared with previous evidence syntheses, the present study differs from both in scope and in the composition of the evidence base. Li et al. [24] performed a conventional pairwise meta-analysis pooling robotic and laparoscopic approaches against open surgery, based on eight studies and 563 patients, with a literature search closing in December 2022; four of their studies (Zhang [10], Rose [6], Beksac [21], Gu et al. [3]) are used in the present review, while four additional studies were published thereafter (Huang et al. [22], Sandberg et al. [23], Gu et al. [7], Tournant et al. [2]), meaning that 591 patients (33% of the present cohort) were not available to that synthesis. Amparore et al. [1] performed a network meta-analysis of 66 studies (3241 patients, largely single-arm), with only seven fully comparative studies informing the NMA cohort and a literature search closing on 10 January 2025; three of the present studies (Sandberg et al. [23], Gu et al. [7], Tournant et al. [2]), contributing 544 patients (31% of the present cohort), postdate that search.

The reduction in estimated blood loss represented the main perioperative advantage of the RARN-TT over ORN-TT and was accompanied by a lower risk of transfusion (Figure 2A,B). Overall, these findings suggest a clinically relevant benefit of the minimally invasive approach in terms of perioperative morbidity. However, this interpretation should be contextualized in light of the substantial heterogeneity across studies. Absolute blood loss varied considerably according to thrombus level, surgical era, and reporting methods [25], whereas transfusion thresholds were rarely defined and depended on local institutional practice. Consequently, the pooled transfusion estimate may conflate the effect of the surgical approach with differences in institutional transfusion practices and CPB-related coagulopathy. Exploratory small-study-effect tests were also nominally significant for transfusion (Egger *p* = 0.0026; Harbord *p* = 0.0001) and, in the same direction, for overall and major complications, although Begg’s test did not confirm this asymmetry for any of the three. With only six contributing studies, these tests are underpowered, but the recurring Egger/Harbord signal—affecting transfusion, one of the few significant findings of this review—warrants some caution in interpreting that benefit as robust. Although the greater precision of dissection provided by the robotic platform may contribute to reducing intraoperative bleeding, the observed benefit may also have been influenced by differences in case complexity, which was generally greater among patients undergoing open surgery than among those treated robotically [26].

Length of hospital stay was the most frequently reported outcome, as it was available in all eight studies, and was significantly shorter after robotic surgery, with a mean reduction of 3.79 days compared with the open approach (Figure 2C). A shorter hospital stay may reduce hospitalization-related resource use and may therefore have potential economic relevance. However, the economic evidence was limited. Gu et al., the only included study reporting a direct economic comparison, found significantly higher mean direct costs for robotic surgery than for open surgery ($12,987 vs. $7337; *p* < 0.001), despite the shorter hospital stay [3]. Therefore, the observed reduction in length of stay cannot be assumed to offset the higher costs associated with the robotic platform. The overall economic sustainability of the robotic approach may depend on several factors, including procedural volume, operative time, institutional experience, platform acquisition and maintenance costs, and the use of disposable instruments. Dedicated cost-effectiveness analyses are needed before firm economic conclusions can be drawn.

Operative time did not differ significantly between approaches but was characterized by marked heterogeneity, indicating substantial variability across studies. This variability may reflect differences in institutional experience, the phase of the learning curve, and the complexity of the cases treated, particularly the distribution of thrombus levels [27]. In a previous matched analysis, median operative time was significantly shorter with the robotic approach than with open surgery [3]. However, other comparative studies reported findings in the opposite direction or no significant differences, without identifying a consistent advantage of either approach in terms of operative duration [28]. Therefore, the absence of an overall difference in the present analysis should be interpreted in light of the technical and clinical variability across centers rather than as evidence of complete equivalence in operative efficiency (Figure 3A).

IVC clamp time was longer with the robotic approach in the only study reporting it (22.2 vs. 13.3 min, a roughly 67% increase), and the lack of comparative data elsewhere precluded a pooled estimate. This difference should not be dismissed as a secondary detail: prolonged caval occlusion can compromise contralateral renal perfusion and lower-limb venous drainage, with potential consequences for intraoperative hemodynamic stability through reduced venous return. Whether this reflects an inherent constraint of the robotic platform or simply the learning curve of the reporting center cannot be established from a single cohort, and remains a question future comparative studies should specifically address.

Overall and major postoperative complications did not differ significantly between the RARN-TT and ORN-TT (Figure 3B,C). Perioperative mortality was also infrequent, and the comparative estimate was imprecise, providing no evidence of a difference between the two approaches (Figure 3D). This outcome was defined as perioperative mortality rather than 90-day mortality because the assessment time point was not consistent across the included studies. Previous evidence syntheses have reported a significant reduction in overall complications with minimally invasive or robotic surgery compared with open surgery, including a decade-long review of robotic series (Garg et al. [28]), a pairwise meta-analysis pooling robotic and laparoscopic approaches (Li et al. [24]: OR 0.33), and a recent network meta-analysis (Amparore et al. [1]: OR 0.37). This reduction in overall complications was not confirmed in the present analysis (RR 0.689, 95% CI 0.411–1.156; *p* = 0.124). The discrepancy may reflect the inclusion of more recent and clinically heterogeneous cohorts not available to these earlier syntheses, differences in the distribution of thrombus levels, and the limited number of events, which reduced the precision of the estimates, and suggests that the earlier finding may not be robust to the inclusion of additional contemporary evidence.

The interpretation of perioperative comparisons is influenced by patient selection. Several robotic cohorts predominantly included Mayo level I–II thrombi, whereas the open surgery groups included a greater proportion of advanced thrombi, including level IV cases that were occasionally managed with cardiopulmonary bypass or intrapericardial control [22]. Because a higher Mayo level is associated with greater surgical complexity and less favorable perioperative outcomes [27], the better outcomes observed with robotic surgery may depend not only on the technical approach but also on the selection of patients with less complex disease. The few available matched studies only partially reduced this risk of confounding.

Beyond patient selection, substantial heterogeneity in operative techniques, both within and between surgical approaches, may have acted as an effect modifier. Robotic procedures encompassed infrahepatic cross-clamping, intrapericardial control without cardiopulmonary bypass, retrohepatic control with the Pringle maneuver, and caval reconstruction using patch angioplasty or segmental grafting, whereas open procedures ranged from midline laparotomy to thoracoabdominal approaches. These technical differences may independently influence operative time, blood loss, transfusion requirements, hemodynamic burden, and postoperative morbidity, thereby limiting the attribution of pooled effects to the surgical approach alone. Because operative details were inconsistently reported and the number of studies was limited, subgroup analyses or meta-regression according to surgical technique could not be performed.

Pathological comparisons did not identify significant differences between robotic and open surgery. However, the limited number of studies and the substantial imprecision of the estimates do not allow true oncological equivalence to be demonstrated, but only the absence of an evident disadvantage associated with the robotic approach [29].

Survival data were limited and were partly reconstructed from Kaplan–Meier curves using the Guyot method [30]. Overall survival did not differ between robotic and open surgery, whereas the other outcomes were based on an insufficient number of studies to support reliable conclusions. Therefore, no evident survival disadvantage emerged for either of the approaches analyzed. Long-term oncological outcomes may also be influenced by systemic therapy, including TKI resistance mechanisms and treatment-related molecular biomarkers, although these factors were outside the scope of the present surgical meta-analysis [31,32].

Evidence regarding the LRN-TT was derived from no more than two studies and, for many outcomes, from a single cohort. These findings should therefore be considered exploratory, including the potential benefit in locoregional recurrence-free survival, which requires confirmation in larger studies.

These findings should be interpreted in light of several limitations. All included studies were retrospective and nonrandomized and had at least a moderate risk of bias, mainly because of confounding and patient selection [33,34]. Specifically, six studies were judged to have a serious risk of bias and one a critical risk, while the only study at moderate risk contributed a relatively small proportion of the overall cohort. A key limitation concerns the Beksac et al. cohort [21], which contributed 838 of the 1411 open cases (59.4%) but did not report thrombus level. Given its substantial weight, the inability to stratify or adjust for thrombus extent may have introduced residual confounding by disease complexity into the pooled estimates. Although the study met the prespecified eligibility criteria, its inclusion warrants particular caution, and the observed associations cannot be considered independent of thrombus level. Together with the sparse and highly imprecise pathological and survival data, this precludes firm conclusions regarding oncological radicality, superiority, or equivalence. The number of studies available for each outcome was limited and did not allow a reliable assessment of publication bias, while the heterogeneity observed for several outcomes reflected differences in thrombus level, surgical era, and reporting methods [35,36]. Furthermore, the inconsistent reporting of relevant variables, including transfusion thresholds, use of cardiopulmonary bypass, and the exact timing of mortality assessment, further limited comparability across studies and the possibility of conducting more detailed analyses.

Thrombus consistency was not included among the variables of interest because previous evidence from a retrospective cohort of 147 patients showed no association between this feature and survival [37]. Nevertheless, its potential relationship with surgical complexity or perioperative outcomes could not be explored, as thrombus consistency was not reported in the included comparative studies.

## 5. Conclusions

The findings of the present analysis are broadly consistent with previous reviews, which reported lower blood loss, reduced transfusion requirements, and a shorter hospital stay after minimally invasive surgery compared with the open approach [1,28,38]. The present meta-analysis updates these findings by including more recent multicenter cohorts and by evaluating robotic, laparoscopic, and open approaches separately within a consistent analytical framework.

In clinical practice, the robotic approach may be considered a reasonable option for appropriately selected patients, particularly those with lower-level thrombi and when surgery is performed at highly experienced centers. The findings suggest lower blood loss and a shorter hospital stay, without evidence of a clear pathological or survival disadvantage. For higher-level thrombi, robotic surgery has proved feasible in selected cases. Still, the available evidence remains insufficient to support its widespread use, and open surgery continues to have an established role [7]. Prospective multicenter studies with stratification by thrombus level and standardized outcome definitions are needed to reduce residual confounding and clarify the role of the different surgical approaches.

## Figures and Tables

**Table 1:** Main characteristics of the studies included in the systematic review and meta-analysis.

Study	Country and Setting	Study Period	RARN-TT, *n*	ORN-TT, *n*	LRN-TT, *n*	Thrombus Eligibility
Gu et al., 2017 [3]	China, single-center	2006–2016	31	37	—	Mayo levels I–II
Beksac et al., 2019 [21]	United States, National Cancer Database	2010–2014	34	838	—	Mayo thrombus level unavailable
Rose et al., 2020 [6]	United States, single-center	1998–2018	24	28	—	Mayo levels I–II
Huang et al., 2023 [22]	China, multicentric	RARN-TT: June 2013–November 2020; ORN-TT: July 2008–November 2020	22	25	—	Mayo Level IV
Zhang et al., 2023 [10]	China, single-center	January 2014–August 2021	22	88	88	Mayo levels 0–IV
Sandberg et al., 2025 [23]	International multicenter study	From 1999; end date NR	23	308	61	Neves levels I–IV
Gu et al., 2025 [7]	China, multicentric	January 2015–December 2023	30	30	—	Mayo levels III–IV
Tournant et al., 2026 [2]	France, eight tertiary referral centers	December 2015–July 2024	35	57	—	Mayo levels I–II
Total			221	1411	149	

**Table 2:** Baseline patient characteristics according to surgical approach.

Outcome	Comparison	Studies, k	Patients, *n*	Effect Estimate (95% CI)	*p* Value	I^2^, %	τ^2^
Male sex	RARN-TT vs. ORN-TT	8	1632	RR 0.997 (0.884–1.125)	0.961	0.0	0.00
	RARN-TT vs. LRN-TT	2	194	RR 0.949 (0.195–4.605)	0.744	0.0	0.00
Age	RARN-TT vs. ORN-TT	8	1632	MD +1.19 yr (−0.86 to +3.25)	0.212	18.2	1.56
	RARN-TT vs. LRN-TT	2	194	MD −0.36 yr (−22.68 to +21.97)	0.873	0.0	0.00
Body mass index	RARN-TT vs. ORN-TT	7	760	MD +0.91 kg/m^2^ (−0.03 to +1.85)	0.056	38.5	0.39
	RARN-TT vs. LRN-TT	2	194	MD +1.29 kg/m^2^ (−22.55 to +25.13)	0.616	85.7	6.11
Charlson Comorbidity Index	RARN-TT vs. ORN-TT	2	399	MD +0.60 (−8.20 to +9.40)	0.547	83.1	0.81
High ASA class	RARN-TT vs. ORN-TT	4	316	RR 0.898 (0.620–1.302)	0.425	0.0	0.00

**Table 3:** Comparative surgical outcomes according to surgical approach.

Outcome	Comparison	Studies, k	Patients, *n*	Effect Estimate (95% CI)	*p* Value	I^2^, %	τ^2^
Operative time	RARN-TT vs. ORN-TT	6	708	MD +13.1 min (−138.9 to +165.2)	0.833	98.1	20,479.9
	RARN-TT vs. LRN-TT	2	194	MD −80.3 min (−1147.9 to +987.3)	0.514	97.8	13,809.9
Estimated blood loss	RARN-TT vs. ORN-TT	6	708	MD −900.5 mL (−1234.0 to −566.9)	0.001	49.4	33,898.3
Blood transfusion	RARN-TT vs. ORN-TT	6	686	RR 0.395 (0.159–0.979)	0.046	86.3	0.47
Cardiopulmonary bypass use	RARN-TT vs. ORN-TT	2	107	RR 0.605 (0.101–3.643)	0.175	0.0	0.00
ICU stay	RARN-TT vs. ORN-TT	3	199	MD +0.43 d (−5.47 to +6.32)	0.785	92.6	5.2
Length of hospital stay	RARN-TT vs. ORN-TT	8	1632	MD −3.79 d (−4.83 to −2.76)	<0.001	40.8	0.51
	RARN-TT vs. LRN-TT	2	194	MD −0.91 d (−9.55 to +7.73)	0.408	0.0	0.0
Overall postoperative complications	RARN-TT vs. ORN-TT	6	429	RR 0.689 (0.411–1.156)	0.124	58.3	0.12
Major postoperative complications	RARN-TT vs. ORN-TT	6	429	RR 0.731 (0.485–1.100)	0.106	0.0	0.00
Perioperative or postoperative mortality	RARN-TT vs. ORN-TT	4	1031	RR 1.601 (0.317–8.088)	0.424	0.0	0.00

**Table 4:** Pathological characteristics according to surgical approach.

Outcome	Comparison	Studies, k	Patients, *n*	Effect Estimate (95% CI)	*p* Value	I^2^, %	τ^2^
Clear-cell RCC histology	RARN-TT vs. ORN-TT	7	746	OR 0.721 (0.375–1.386)	0.267	0.0	0.00
	RARN-TT vs. LRN-TT	2	193	OR 1.166 (0.001–908.933)	0.818	0.0	0.00
High-grade tumor	RARN-TT vs. ORN-TT	6	653	OR 0.729 (0.347–1.532)	0.323	7.4	0.00
Positive lymph nodes	RARN-TT vs. ORN-TT	3	264	OR 0.707 (0.170–2.948)	0.406	0.0	0.00

**Table 5:** Survival outcomes comparing RARN-TT and ORN-TT.

Outcome	Comparison	Studies, k	Pooled HR (95% CI)	*p* Value	I^2^, %	τ^2^
Overall survival	RARN-TT vs. ORN-TT	4	0.877 (0.444–1.733)	0.583	28.5	0.0397
Cancer-specific survival	RARN-TT vs. ORN-TT	2	1.031 (0.009–114.401)	0.948	20.6	0.0699
Progression-free survival	RARN-TT vs. ORN-TT	2	0.961 (0.111–8.359)	0.855	0.0	0.0000

## Data Availability

The data presented in this study are available in the article and its Supplementary Materials. Further data extracted from the included studies are available from the corresponding author upon reasonable request.

## Supplementary Materials

The following supporting information can be downloaded at: https://www.mdpi.com/2072-6694/18/16/2613/s1, Supplementary Table S1: Baseline characteristics; Supplementary Table S2: Surgical outcomes; Supplementary Table S3: Pathological outcomes; Supplementary Table S4: Survival outcomes; Supplementary Table S5: Small-study effect tests.
