# Supplementary material for: Robot-Assisted Versus Open and Laparoscopic Radical Nephrectomy with Inferior Vena Cava Thrombectomy for Renal Cell Carcinoma: A Systematic Review and Meta-Analysis
Source: Cancers (Basel). 2026 Aug 13;18(16):2613. doi: 10.3390/cancers18162613 (PMC13511196; doi:10.3390/cancers18162613)
Supplement: Supplementary file 1 [file cancers-18-02613-s001.zip › cancers-4429047-supplementary.pdf]

Supplementary Table S1: Baseline characteristics

Variable	Comparison	Studies, k	RARN-TT, n	Comparator, n	Effect estimate (95% CI)	p value	Cochran Q (df)	p for heterogeneity	I^2, %	τ^2	95% prediction interval
Male sex	RARN-TT vs ORN-TT	8	221	1,411	RR 0.997 (0.884–1.125)	0.961	5.17 (7)	0.639	0.0	0.0000	0.880–1.129
Male sex	RARN-TT vs LRN-TT	2	45	149	RR 0.949 (0.195–4.605)	0.744	0.16 (1)	0.686	0.0	0.0000	Not calculated
Age	RARN-TT vs ORN-TT	8	221	1,411	MD +1.193 yr (−0.863 to +3.249)	0.212	8.55 (7)	0.286	18.2	1.5603	−2.531 to +4.917 yr
Age	RARN-TT vs LRN-TT	2	45	149	MD −0.356 yr (−22.684 to +21.972)	0.873	0.13 (1)	0.714	0.0	0.0000	Not calculated
Body mass index	RARN-TT vs ORN-TT	7	187	573	MD +0.909 kg/m² (−0.032 to +1.850)	0.056	9.75 (6)	0.135	38.5	0.3858	−0.969 to +2.787 kg/m²
Body mass index	RARN-TT vs LRN-TT	2	45	149	MD +1.293 kg/m² (−22.549 to +25.134)	0.616	7.01 (1)	0.008	85.7	6.1094	Not calculated
Charlson Comorbidity Index	RARN-TT vs ORN-TT	2	54	345	MD +0.597 (−8.201 to +9.395)	0.547	5.92 (1)	0.015	83.1	0.8146	Not calculated
High ASA class	RARN-TT vs ORN-TT	4	106	210	RR 0.898 (0.620–1.302)	0.425	0.30 (3)	0.959	0.0	0.0000	0.544–1.483
High ASA class	RARN-TT vs LRN-TT	1	22	88	RR 1.333 (0.394–4.517)	Not pooled	NA	NA	NA	NA	Not calculated

Abbreviations: ASA = American Society of Anesthesiologists; CI = confidence interval; df = degrees of freedom; LRN-TT = laparoscopic radical nephrectomy with tumor thrombectomy; MD = mean difference; NA = not applicable; ORN-TT = open radical nephrectomy with tumor thrombectomy; RARN-TT = robot assisted radical nephrectomy with tumor thrombectomy; RR = risk ratio.

Supplementary Table S2: Surgical outcomes

Panel A. Primary comparative analyses

Outcome	Comparison	Studies, k	Analytic sample, RARN-TT/comparator	Effect estimate (95% CI)	p value	Cochran Q (df)	p for heterogeneity	I², %	τ²	95% prediction interval
Operative time	RARN-TT vs ORN-TT	6	163/545	MD +13.1 min (−138.9 to +165.2)	0.833	267.79 (5)	<0.001	98.1	20,479.9 min²	−416.8 to +443.1 min
Operative time	RARN-TT vs LRN-TT	2	45/149	MD −80.3 min (−1147.9 to +987.3)	0.514	45.47 (1)	<0.001	97.8	13,809.9 min²	Not calculated
Estimated blood loss	RARN-TT vs ORN-TT	6	163/545	MD −900.5 mL (−1234.0 to −566.9)	0.001	9.88 (5)	0.079	49.4	33,898.3 mL²	−1525.8 to −275.1 mL
Blood transfusion	RARN-TT vs ORN-TT	6	155/531	RR 0.395 (0.159–0.979)	0.046	36.58 (5)	<0.001	86.3	0.4765	RR 0.046–3.399
Cardiopulmonary bypass use	RARN-TT vs ORN-TT	2 estimable	52/55	RR 0.605 (0.101–3.643)	0.175	0.58 (1)	0.448	0.0	0.0000	Not calculated
ICU stay	RARN-TT vs ORN-TT	3	87/112	MD +0.43 d (−5.47 to +6.32)	0.785	27.18 (2)	<0.001	92.6	5.2007 d²	−33.37 to +34.22 d
Length of hospital stay	RARN-TT vs ORN-TT	8	221/1411	MD −3.79 d (−4.83 to −2.76)	<0.001	11.83 (7)	0.106	40.8	0.5130 d²	−5.85 to −1.74 d
Length of hospital stay	RARN-TT vs LRN-TT	2	45/149	MD −0.91 d (−9.55 to +7.73)	0.408	0.21 (1)	0.645	0.0	0.0000	Not calculated
Overall postoperative complications	RARN-TT vs ORN-TT	6	164/265	RR 0.689 (0.411–1.156)	0.124	11.99 (5)	0.035	58.3	0.1277	RR 0.221–2.152
Major postoperative complications	RARN-TT vs ORN-TT	6	164/265	RR 0.731 (0.485–1.100)	0.106	2.10 (5)	0.836	0.0	0.0000	RR 0.470–1.137
Perioperative or postoperative mortality	RARN-TT vs ORN-TT	4 estimable	110/921	RR 1.601 (0.317–8.088)	0.424	1.88 (3)	0.597	0.0	0.0000	RR 0.179–14.304

Panel B. Sensitivity and additional pooled analyses

Analysis	Studies, k	Patients, n	Effect estimate (95% CI)	p value	Cochran Q (df)	p for heterogeneity	I², %	τ²
Overall postoperative complications, excluding Huang et al.	5	382	RR 0.570 (0.280–1.160)	0.093	8.38 (4)	0.078	52.3	0.1521
Conversion to open surgery within RARN-TT cohorts	4	121	7% (3–13%)	NA	0.57 (3)	0.900	0.0	0.0000

Panel C. Outcomes not included in quantitative comparative synthesis

Outcome	Comparison	Study or evidence base	RARN-TT result	Comparator result	Reason not pooled
IVC clamp time	RARN-TT vs ORN-TT	Tournant et al.	Mean 22.2 min	Mean 13.3 min	One study with complete comparative data
Estimated blood loss	RARN-TT vs LRN-TT	Zhang et al.	Mean 234.5 mL	Mean 505.0 mL	One study only
Blood transfusion	RARN-TT vs LRN-TT	Zhang et al.	8/22 (36.4%)	38/88 (43.2%)	One study only
Overall postoperative complications	RARN-TT vs LRN-TT	Zhang et al.	4/22 (18.2%)	20/88 (22.7%)	One study only
Major postoperative complications	RARN-TT vs LRN-TT	Zhang et al.	0/22 (0%)	4/88 (4.5%)	One study only
Conversion to open surgery	RARN-TT vs LRN-TT	Zhang et al.	1/22 (4.5%)	8/88 (9.1%)	One study only

Abbreviations: CI = confidence interval; df = degrees of freedom; ICU = intensive care unit; LRN-TT = laparoscopic radical nephrectomy with tumor thrombectomy; MD = mean difference; NA = not applicable; ORN-TT = open radical nephrectomy with tumor thrombectomy; RARN-TT = robot assisted radical nephrectomy with tumor thrombectomy; RR = risk ratio.

Supplementary Table S3: Pathological outcomes

Panel A. Pooled comparative analyses

Outcome	Comparison	Studies, k	RARN-TT, n	Comparator, n	OR (95% CI)	p value	Cochran Q (df)	p for heterogeneity	I², %	τ²
Clear-cell RCC histology	RARN-TT vs ORN-TT	7	187	559	0.721 (0.375–1.386)	0.267	3.85 (6)	0.696	0.0	0.0000
Clear-cell RCC histology	RARN-TT vs LRN-TT	2	45	148	1.166 (0.001–908.933)	0.818	0.79 (1)	0.374	0.0	0.0000
Positive lymph nodes	RARN-TT vs ORN-TT	3	88	176	0.707 (0.170–2.948)	0.406	0.72 (2)	0.696	0.0	0.0000
High-grade tumor	RARN-TT vs ORN-TT	6	142	511	0.729 (0.347–1.532)	0.323	5.40 (5)	0.369	7.4	0.0000
High-grade tumor	RARN-TT vs LRN-TT	2	33	144	0.795 (0.002–261.587)	0.703	0.50 (1)	0.479	0.0	0.0000

Panel B. Outcomes not included in quantitative comparative synthesis

Outcome	Comparison	Study coverage	RARN-TT result	Comparator result	Reason not pooled
Advanced pathological stage	RARN-TT vs ORN-TT	4 studies	105/105 (100%)	448/448 (100%)	No variation within either treatment group; comparative OR not estimable
Advanced pathological stage	RARN-TT vs LRN-TT	2 studies	44/44 (100%)	145/146 (99.3%)	Near-universal events and only one informative study
IVC wall invasion	RARN-TT vs LRN-TT	Zhang et al.	4/22 (18.2%)	21/88 (23.9%)	One study only
IVC wall invasion	RARN-TT vs ORN-TT	2 studies	30% (95% CI 18–44%)	26% (95% CI 18–35%)	Two studies only, with discordant study-specific effects; comparative OR spanned several orders of magnitude (95% CI 0.000–4668.760) and was not informative
Positive lymph nodes	RARN-TT vs LRN-TT	Zhang et al.	13/22 (59.1%)	54/88 (61.4%)	One study only
Positive surgical margins	RARN-TT vs ORN-TT	2 studies	6% (95% CI 1–14%)	20% (95% CI 12–30%)	Two studies only, with discordant study-specific effects; comparative OR spanned several orders of magnitude (95% CI 0.000–343.023) and was not informative
Positive surgical margins	RARN-TT vs LRN-TT	No complete comparative data	-	-	Complete comparative data unavailable

Abbreviations: CI = confidence interval; df = degrees of freedom; IVC = inferior vena cava; LRN-TT = laparoscopic radical nephrectomy with tumor thrombectomy; NR = not reported; OR = odds ratio; ORN-TT = open radical nephrectomy with tumor thrombectomy; RARN-TT = robot assisted radical nephrectomy with tumor thrombectomy; RCC = renal cell carcinoma.

Supplementary Table S4: Survival outcomes

Panel A. Pooled comparative analyses

Outcome	Comparison	Studies, k	Pooled HR (95% CI)	p value	Cochran Q (df)	p for heterogeneity	I^2, %	τ^2	95% Prediction interval
Overall survival	RARN-TT vs ORN-TT	4	0.877 (0.444–1.733)	0.583	4.19 (3)	0.241	28.5	0.0397	0.249–3.085
Cancer-specific survival	RARN-TT vs ORN-TT	2	1.031 (0.009–114.401)	0.948	1.26 (1)	0.262	20.6	0.0699	Not calculated (k=2)
Progression-free survival	RARN-TT vs ORN-TT	2	0.961 (0.111–8.359)	0.855	0.04 (1)	0.842	0.0	0.0000	Not calculated (k=2)

Panel B. Outcomes not included in quantitative comparative synthesis

Outcome	Comparison	Study	HR (95% CI)	Estimate source
RFS	RARN-TT vs ORN-TT	Tournant et al.	0.440 (0.180–1.070)	Direct Cox HR
DFS	RARN-TT vs ORN-TT	Tournant et al.	0.500 (0.220–1.120)	Direct Cox HR
MFS	RARN-TT vs ORN-TT	Sandberg et al.	1.882 (0.900–3.937)	Reconstructed from KM curves
OS	LRN-TT vs ORN-TT	Sandberg et al.	1.152 (0.751–1.766)	Reconstructed from KM curves
OS	RARN-TT vs LRN-TT	Sandberg et al.	0.965 (0.474–1.964)	Reconstructed from KM curves
CSS	LRN-TT vs ORN-TT	Sandberg et al.	0.889 (0.516–1.530)	Reconstructed from KM curves
CSS	RARN-TT vs LRN-TT	Sandberg et al.	1.461 (0.639–3.344)	Reconstructed from KM curves
MFS	LRN-TT vs ORN-TT	Sandberg et al.	1.334 (0.791–2.249)	Reconstructed from KM curves
MFS	RARN-TT vs LRN-TT	Sandberg et al.	1.473 (0.627–3.456)	Reconstructed from KM curves
OS	Minimally invasive vs ORN-TT	Zhang et al.	1.200 (0.810–1.770)	Direct Cox HR
CSS	Minimally invasive vs ORN-TT	Zhang et al.	1.170 (0.780–1.770)	Direct Cox HR
MFS	Minimally invasive vs ORN-TT	Zhang et al.	0.980 (0.640–1.510)	Direct Cox HR
Locoregional RFS	Minimally invasive vs ORN-TT	Zhang et al.	0.200 (0.060–0.700)	Direct Cox HR

Abbreviations: CI = confidence interval; CSS = cancer-specific survival; DFS = disease-free survival; HR = hazard ratio; KM = Kaplan–Meier; LRN-TT = laparoscopic radical nephrectomy with tumor thrombectomy; MFS = metastasis-free survival; ORN-TT = open radical nephrectomy with tumor thrombectomy; OS = overall survival; RARN-TT = robot assisted radical nephrectomy with tumor thrombectomy; RFS = recurrence-free survival.

Supplementary Table S5: Small-study effect tests

Outcome category	Outcome	Comparison	Effect measure	Studies, k	Egger coefficient; p value	Begg z; p value	Harbord coefficient; p value
**Baseline characteristics**	Age	RARN-TT vs ORN-TT	MD	8	−1.29; 0.0475	−1.61; 0.1735	-
	Body mass index	RARN-TT vs ORN-TT	MD	7	−0.03; 0.9870	0.30; 0.7639	-
	Male sex	RARN-TT vs ORN-TT	RR	8	−1.73; 0.3421	−0.37; 0.9015	−0.58; 0.7590
**Surgical outcomes**	Operative time	RARN-TT vs ORN-TT	MD	6	25.06; 0.0547	2.25; 0.0242	-
	Estimated blood loss	RARN-TT vs ORN-TT	MD	6	1.78; 0.2396	0.00; 1.0000	-
	ICU stay	RARN-TT vs ORN-TT	MD	3	24.84; 0.1752	1.04; 0.2963	-
	Length of hospital stay	RARN-TT vs ORN-TT	MD	8	−0.32; 0.8047	0.12; 0.9015	-
	Blood transfusion	RARN-TT vs ORN-TT	RR	6	−3.40; 0.0026	−1.13; 0.4524	−6.15; 0.0001
	Overall postoperative complications	RARN-TT vs ORN-TT	RR	6	−1.80; 0.0031	−1.50; 0.2597	−1.79; 0.0121
	Major postoperative complications	RARN-TT vs ORN-TT	RR	6	−0.90; 0.0025	−1.13; 0.4524	−1.09; 0.0009
	Perioperative or postoperative mortality	RARN-TT vs ORN-TT	RR	4	−2.02; 0.1221	−1.70; 0.3082	−1.59; 0.5654
**Pathological outcomes**	Clear cell renal cell carcinoma	RARN-TT vs ORN-TT	OR	7	2.32; 0.1427	1.20; 0.2296	0.18; 0.9621
	High grade tumor	RARN-TT vs ORN-TT	OR	6	−1.62; 0.4337	−1.50; 0.2597	−1.48; 0.6054
	Positive surgical margins	RARN-TT vs ORN-TT	OR	2	-	-	-
	Positive lymph nodes	RARN-TT vs ORN-TT	OR	3	−2.02; 0.2178	−1.04; 1.0000	−2.41; 0.1982
**Oncological outcomes**	Overall survival	RARN-TT vs ORN-TT	HR	4	−0.24; 0.9568	−0.34; 1.0000	-

Egger and Harbord results are presented as regression coefficients with corresponding p values. Begg results are presented as standardized z statistics with corresponding p values. All tests were exploratory because every analysis included fewer than ten studies. Therefore, nominally significant results should not be interpreted as definitive evidence of publication bias, particularly in view of the limited statistical power and the number of tests performed. Harbord testing was applied only to binary outcomes. Tests were not estimated when fewer than three informative studies were available.

Abbreviations: HR = hazard ratio; ICU = intensive care unit; MD = mean difference; NA = not applicable; NE = not estimated; OR = odds ratio; ORN-TT = open radical nephrectomy with tumor thrombectomy; RARN-TT = robot assisted radical nephrectomy with tumor thrombectomy; RR = risk ratio.
